# Iran’s health insurance ecosystem: challenges and strategies

**DOI:** 10.1186/s12889-024-19998-2

**Published:** 2024-09-11

**Authors:** Rohaneh Rahimisadegh, Somayeh Noori Hekmat, Mohammad Hossein Mehrolhassani, Mohammad Jafari Sirizi

**Affiliations:** 1 Health Services Management Research Center, Institute for Futures Studies in Health, Kerman University of Medical Sciences, Kerman, Iran; 2 Health Foresight and Innovation Research Center, Institute for Futures Studies in Health, Kerman University of Medical Sciences, Kerman, Iran; 3 Department of Health Management, Policy and Economics, Faculty of Management and Medical Information Sciences, Kerman University of Medical Sciences, Kerman, Iran; 4 National Center for Health Insurance Research, Tehran, Iran

**Keywords:** Ecosystem, Health insurance, Stewardship and financing functions, Challenge, Strategy

## Abstract

**Introduction:**

Identifying and exploiting stewardship and financing challenges in Iran’s health insurance system as an ecosystem is essential to achieving predetermined goals. This study aimed to determine the challenges and strategies in the Iranian health insurance ecosystem to provide relevant evidence to healthcare managers and policymakers to improve its functions and perform necessary reforms.

**Method:**

This qualitative study was conducted at the national level in Iran. Data were collected using semi-structured interviews and analyzed using the directed content analysis method. The study participants included managers and experts in health insurance and faculty of universities of medical sciences, who were selected by purposive sampling.

**Results:**

The challenges and strategies expressed by participants were categorized into two functions: stewardship and financing. Four main themes, ten subthemes, 22 challenges, and 24 strategies were identified in the stewardship function, along with three main themes, 12 subthemes, 17 challenges, and 16 strategies in the financing function. The major challenge in the Iranian health insurance ecosystem was the complexity and conflict of interests between multiple actors with different roles, which led to fragmentation, diverse structures, and a gap between other functions and objectives, hindering the effective functioning of the ecosystem.

**Conclusion:**

In order to deal with the challenges of the health insurance ecosystem, it is suggested to create a coherent insurance system through a single utility system, and by paying more attention to health-oriented services, the health insurance ecosystem becomes a health-oriented system instead of being treatment-oriented. In addition, in order to strengthen the governance of the country’s health insurance ecosystem, the number of actors with multiple roles should be reduced and the roles of the actors should be clarified and separated in order to prevent conflicts of interest and structural corruption in this ecosystem.

**Supplementary Information:**

The online version contains supplementary material available at 10.1186/s12889-024-19998-2.

## Introduction

In recent years, the concept of the ecosystem has been increasingly used to interpret competitive environments and new businesses [1]. The business ecosystem approach is an essential and new theory in strategic management [2], first proposed by James Moore (1993) [3]. Moore defines the business ecosystem as “an economic community that arises from the interaction of organizations, individuals, and other components of the business world.” According to this definition, the ecosystem includes various actors [4] with mutual relationships and interactions [5]. Business ecosystem theory systematically describes business environments and systematically analyzes the effects of decisions made by different organizations and companies on each other [6]. In recent years, new actors have entered the ecosystem in large businesses such as insurance and captured part of the market or replaced some existing actors from the market [7]. In such a situation, insurance ecosystem actors, including insurance organizations, are forced to adopt newer strategies and solutions to succeed, survive, and maintain their position against existing challenges [8–10].

In Iran, the health system is funded through a mixed budget system and is primarily based on social security. Health insurance is funded from four main sources: tax revenues, oil sales revenues, out-of-pocket expenditure, and insurance contributions [11]. The Universal Health Insurance (UHI) law was drafted in 1992 to address the issue of increasing health costs after the Iran-Iraq war. The UHI Law aimed to cover 60% of Iran’s uninsured population, and the Medical Services Insurance Organization (MSIO) was established with the goal of covering a broad range of the population within five years [12]. Before the UHI Law was enacted, there was no clear law regarding insurance companies and only mentioned the compensation of benefits. The passing of the UHI Law and the subsequent establishment of the MSIO have brought about the desired cohesion and consistency between Iran’s health insurance policies and laws [13]. In Iran, the Ministry of Health and Medical Education (MOHME) plays the primary role as both buyer and supplier, serving the majority of the population. In addition, there are many other actors in the public and private sectors who are also buyers, financiers, and service providers. The payment system is considered a complex payment system with multiple flows of funds from different sources and using different methods to public and private providers [14]. Iran spent 6.7% of its GDP on health, according to WHO of National Health Accounts data for 2019. The report said the main sources of funding for health expenditure were basic health insurance and contributions from the state budget, accounting for 49.5%, and out-of-pocket payments for 39.5% [15]. Iran’s health insurance system includes basic health insurance organizations such as Iran Health Insurance, Social Security, Armed Forces Medical Services Insurance Organization (AFMSIO), and other health insurance companies affiliated with banks and other institutions responsible for providing basic health services to the insured. In addition, some companies offer supplementary health insurance that covers types of medical services not covered by basic health insurance [16], which has always included challenges in this direction. Because of the requirements of Iran’s 5th and 6th Development Plans, it is of great importance to reduce out-of-pocket expenses, increase the government’s share in financing public health expenditures, and properly allocate public funds to the health sector. For this reason, providing equitable and accessible health services to the public has become one of the concerns of governments of various countries and achieving this goal always comes with serious challenges [17]. The Health System Transformation Plan was implemented in 2012 to improve access to medical services for needy people and increase the proportion of society covered by insurance. Accordingly, uninsured people received free health insurance. Since the financial resources required to implement this plan were not provided sustainably, the continuation became a significant challenge for the Iran Health Insurance Organization (IHIO) and placed severe financial pressure on this organization [18]. Another fundamental problem of the health insurance system is the large number of insurance carriers and organizations. Although several years have passed since the adoption and implementation of the consolidation of health insurance funds law, this law has not yet been implemented [19].

In addition to the mentioned challenges, other challenges have also been identified in the studies. In this regard, the studies conducted in this regard can be divided into two categories. The first category are studies that have examined the challenges in a specific issue in the health insurance system from a thematic perspective, issues such as strategic purchase, coverage, etc [20–23]. The second category are studies that have identified challenges in one of the country’s health insurance organizations, such as the health insurance organization or the social security insurance organization, as one of the main players in the health insurance ecosystem [24]. In addition to these two types of studies, a study was conducted by Davari et al. in 2012, which identified the challenges of the health insurance system in Iran with a broader perspective [12].Considering that more than 10 years have passed since the study by Davari et al. and in recent years we have seen many changes in the elements, actors and interactions of Iran’s health insurance ecosystem, therefore, it is necessary to conduct a study from the perspective of the ecosystem, which identifies the challenges of Iran’s health insurance system in a macro and comprehensive perspective, and the present study was carried out in response to this necessity.

## Method

### Study design

**Fig. 1:**
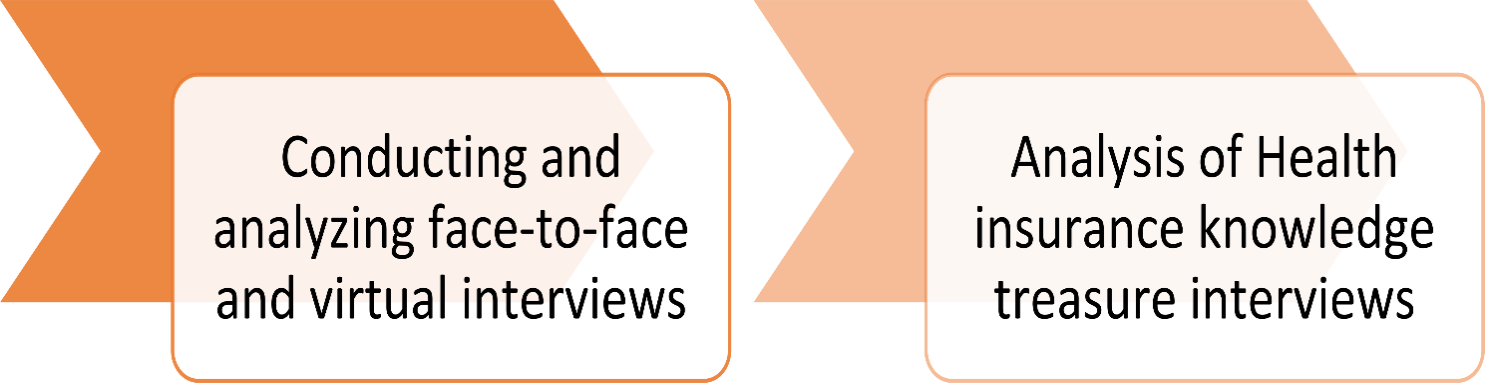
Study stages

This qualitative study aimed to identify the challenges of Iran’s health insurance ecosystem and the related strategies in 2021 in two phases (Fig. 1).

### Participants

Participants in the study included medical sciences university faculty members, managers, and health insurance industry experts who were selected through purposive and snowball sampling with maximum heterogeneity samples. Participants were required to have a solid academic and scientific background in health insurance or over five years of executive experience. Table 1 shows the characteristics of the study participants. The information also encompasses participants who participated in Health insurance knowledge treasure interviews regarding health insurance. Further details about these interviews can be found in the data collection section.

**Table 1:** Characteristics of study participants

The general category	Characteristics	No.
**Gender**	Female	15
	Male	41
**Organizational position**	Expert	8
	Manager	41
	Faculty	7
**Geographical scale**	Provincial	24
	National	32
**Type of interview**	Virtual interviews	4
	Face-to-face interviews	24
	Health insurance knowledge treasure interviews	28
**Total**		56

### Data collection

A semi-structured interview was used as the data collection tool. The research team created the interview framework with the study objective. There were two sections to the interview framework questions. Participants were questioned about the challenges facing the insurance ecosystem in the first section and the related strategies in the second. Since this study was conducted during the COVID-19 outbreak because of interprovincial travel restrictions, 24 face-to-face and four virtual interviews were conducted. Face-to-face and virtual interviews were conducted until data saturation was reached (RR conducted the interview). Face-to-face interviews were conducted at participants’ workplaces, and virtual interviews were conducted through WhatsApp. Participants were informed of the purpose of the research before completing each interview, and all face-to-face interviews were recorded with each individual’s permission. In one interview, one participant did not allow the voice to be recorded, and the researcher noted all topics raised in the interview. The researcher also took essential notes during all the interviews.

### Data validity and reliability

The triangulation method was used to increase the validity of the data [25]. Thus, the data from the interviews under the title “Health insurance knowledge treasure” conducted by the National Health Insurance Research Center with experienced managers and experts in health insurance were utilized besides the face-to-face and virtual interviews. In these interviews, managers and experts who had executive and managerial experience in various organizations in the health insurance ecosystem expressed challenges, and their data helped enrich and increase the validity of the data collected from face-to-face and virtual interviews. The Guba and Lincoln indices, which include the concepts of credibility, transferability, verifiability, and reliability, were used to verify the accuracy and robustness of the data [26].

### Data analysis

The directed content analysis was used to analyze the interviews. RR and MHM read the implemented text of the interviews separately and extracted codes according to the health system functional model with a comparative approach (in this study, only stewardship and financing were relevant) [27]. When researchers disagreed about the codes created, the differences were reviewed and compared in joint meetings, and the differences were discussed until a consensus was reached. This process led to forming key themes and sub-themes and provided a framework for interpreting the results. The MAXQDA2020 software was applied to code the interviews. All steps for coding face-to-face and virtual interviews were also performed for the health insurance knowledge treasure interviews, with the difference that the text of these interviews was available on the National Center for Health Insurance Research website. There was no need to implement their text.

## Results

The challenges and strategies expressed by the participants regarding the two functions of managing and financing the health system are presented separately in Tables 2 and 3.

### Stewardship function

According to Table 2, four main themes, ten subthemes, 22 challenges, and 24 strategies were identified in the function of the stewardship of the health insurance ecosystem. The following are participants’ opinions on the essential challenges and strategies.

#### Challenges

***Lack of stability in the structure and stewardship of the health insurance system***

*“Previously*,* we were initially under the supervision of the Ministry of Health for the last 25 years. After ten years*,* we were under the supervision of the Ministry of Social Affairs*,* and after another ten years*,* we returned to the Ministry of Health. We have experienced it many times in different structures” (P: 35).*

***The multiplicity of insurance funds***

*“The mistake was that we broke the insurance back into pieces*,* whereas the insurance is stronger. The insurance system has power worldwide*,* but we have blocked that power by fragmenting insurance” (P: 4).*

***Lack of health-oriented attitude***

*“I said that you should mention the insured position in your mission*,* and your mission is to provide health services. They said our mission is only treatment*,* and we cannot enter health. I said why? He said because the law mentions treatment” (P: 43).*

***Traditional and non-flexible structures***

*“What I see in the organization in recent years as the bane of our human lives is routine. Any organization that falls into a routine means it will collapse. An organization that could be very dynamic has become an everyday organization” (P: 10).*

***Lack of transparency of the rules of insurance organizations***

*“Armed Forces Health Insurance is always problematic*,* and it is unclear what laws they follow and according to which laws they deduct insurance payments.” (P: 52).*

***Conflict of interest due to the multiple roles of the actors***

*“The government share on the board of social security trustees has increased a lot. Why? Because he took the other two sides*,* the employer*,* and the workers*,* and introduced them from the ministry” (P: 48).*

***Lack of coordination***,*** cooperation and constructive interaction***

*“Supplementary medical insurance*,* which makes up a significant portion of our insurances*,* requires coordination with basic health insurance” (P: 12).*

***Sectional and personal decision-making***

*“Sectional and personal decisions are one case that does not place the health insurance organization in its real position “(P: 8).*

***The narrow vision of the Ministry of Health in the stewardship***

*“A person who is placed in health-oriented organizations and wants to make decisions in the social field should not have gun barrel thinking (limited thinking) but should have a comprehensive view and high perceptive capabilities” (P: 10).*

***The existence of multiple and separate information systems***

*“Once we made a list of the software and information systems in the headquarters and treatment management. About thirty pieces of software were not linked to each other. This means that when we need data*,* we have to search for it in different systems” (P: 43).*

**Table 2:** Challenges and strategies in stewardship function of Iran’s health insurance ecosystem

Main theme	Subtheme	Challenges	Strategies
**Governance-structure**	Mission and existential philosophy	• Misunderstanding of some managers and policymakers about the function and principles of insurance	• Creating a holistic and health-oriented attitude and coverage of preventive level services in the insurance system •Prioritizing health as one of the basic priorities of governments • The need to change the paradigm, mission and paradigm shift in the insurance system
	Structure	• Lack of stability in the structure and stewardship of the health insurance system • The multiplicity of insurance funds • Traditional and non-flexible structures	• Reviewing the organizational structure of health insurance and creating a stable and independent structure from the ministerial structure • Consolidation of insurance funds and unification of insurance policies • Agility of the structure of the insurance system by decentralizing the duties of the governing bodies
**Governance-legislation**	Regulatory mechanisms such as rules and regulations	• Failure to fully implement insurance guidelines and laws by insurance organizations • Lack of transparency of the rules of insurance organizations • Lack of commitment of governance and administration to implement laws	• Establishing the requirement for the full implementation of the law of general insurance of medical services of the country by all insurance organizations
	Monitoring and evaluation	• Weak monitoring of the performance of service providers by governing bodies	• Using the insurance system as a cost control tool
	Punitive and incentive mechanisms	• Moral hazard and induced demand	• Applying a cap on the receipt of high-use medical services in order to control and correct the consumption pattern
**Interdepartmental leadership and coordination**	Determining positions	• Conflict of interests due to the multiple roles of the actors • Collusion and lobbying	• Separation of political games from the country’s insurance system • Transparency in the duties of actors with multiple roles
	Intersectional communication and cooperation	• Lack of coordination, cooperation and constructive interaction between the ecosystem actors of organizations in order to establish effective communication	• Improving relations and cooperation and creating alignment in interests • Strengthening the Insurance Coordinating Council in order to expand cooperation and alignment
	Human resources	• Non-compliance with professional ethics by some service providers • Appointment of specialist and specialist doctors in management positions	• Empowering presenters regarding the principles of professional ethics • Empowering doctors regarding management principles and techniques
**Policy making**	Rational decision making	• The sectional and personal decision-making • The narrow vision of the Ministry of Health in the stewardship of the health system as an obstacle to macro and intersectoral decisions • Failure to use scientific and research results and evidence in executive and policy-making actions	• Avoiding hasty decision-making and using decision-making and policy-making based on collective wisdom and scientific evidence • Establishing a monitoring and data mining system in the insurance system • Conducting scientific and comparative studies in the field of insurance to produce evidence
	Information Resources	•The existence of multiple and separate information systems • The resistance of service providers against electronicization of the insurance system and • Weakness in electronics infrastructure • Lack of transparency in the executive processes of electronicization and systems • Application of hospital deductions due to users’ unfamiliarity with the relevant systems	• Implementation of the electronic prescribing plan completely •Unification and integration of information systems • Improvement of information technology infrastructure • Creating an online database and collecting information from insurance organizations • Defining rules in the systems to prevent errors and violations and create transparency • Forming an electronic health record

#### Strategies

***Creating a holistic and health-oriented attitude***

*“A multifaceted view is fundamental in health issues*,* especially for an organization that has changed from medical service insurance to health insurance. Perhaps many parts*,* including rehabilitation*,* are not part of the insurance obligations*,* but health is a comprehensive word*,* and we must make more efforts to realize it” (P: 3).*

***Consolidation of insurance funds***

*“I believe that there is no need to unify all insurances because there are countries where all insurances are not the same*,* and they have good insurance*,* such as the Netherlands*,* where the basic insurances have not been unified*,* but they follow a single policy” (P:7).*

***Improving relations and cooperation and creating alignment in interests***

*“If the social security organization cooperates with us*,* we can assign the insured population to the doctors on the contract side and realize the leveling of services to receive more and better services by our insured” (P:2).*

***Decision-making and policy-making are based on collective wisdom and scientific evidence.***

*“This data mining should have several specific outputs. One output is to become a decision support system that helps make the right decision. We cannot decide until we have the right data” (P: 10).*

***Implementation of the electronic prescribing plan completely***

*“It would be wonderful if*,* like advanced countries*,* a comprehensive electronic prescription system is completed and paper prescriptions are eliminated in all pharmacies and laboratories” (P: 23).*

***Unification and integration of information systems***

*“We had a company that contracted with the Ministry of Welfare to data mine our information. Unfortunately*,* our information sources were scattered and insular. Our information sources were in such a way that each province entered information separately. In one province*,* code 2 to the pharmacy was given code 3 in another province. This company had standardized all the codes and gathered the information for us without our provinces realizing it” (P: 9).*

***Forming an electronic health record***

*“Day and night*,* I followed the discussion of setting up electronic records and electronic health insurance so that the health system’s information would be in front of my eyes like a dashboard. As a result*,* I could figure out how much money a doctor generates for insurance and this cost*,* and if the cost was higher*,* I could have quickly informed him he was no longer a party to the insurance contract because of the unreasonable prescription of medicine or service” (P:20).*

### Financing function

Table 3 identifies three main themes, 12 subthemes, 17 challenges, and 16 strategies in the financing function. In the following, a sample of the interviewees’ opinions is expressed.

#### Challenges

***Imbalance between cost and income of insurance organizations***

*“The problem is that the organization’s resources have never equaled the costs. The resources and costs are not consistent with each other*,* so the organization is always faced with accumulated losses and a lack of balance in the resources and costs. People and government resources are provided*,* public resources should be proportional to the people’s ability to pay*,* and government resources usually face a shortage. On the other hand*,* the expenses are determined based on the needs of the insured and the scientific methods of doctors and medical service providers. The resources are calculated*,* estimated*,* and funded in one way*,* and our costs are determined in another way*,* which makes the insurance system a challenge” (P: 19).*

***Multi-tariff services***

*“The insured can get medical services in several departments; we have multiple tariffs. One of them is the university’s government department*,* and our institutions are divided into four departments” (P: 44).*

***Unreality of service prices and tariffs in the insurance system***

*“In the universal insurance law*,* it was foreseen that the government would set a tariff*,* but if the tariff is lower than the real price*,* it will compensate for the difference. The tariff will be determined based on the people’s pockets*,* economic conditions of the country*,* and the real price*,* but are the tariffs is currently determined on the same basis? (This case deserves criticism).” (P: 8).*

***Insurance deductions***

*“The insurance expert makes a deduction on the hospitalization file and sends the file to the revenue unit. The head of the revenue unit looks at the file and approves if they accept the expert’s deductions. The file is returned to the insurance expert if they do not accept the expert’s deductions. The expert re-examines the file” (P: 32).*

***Overlap of the coverage population***

*“The next point is overlaps*,* which have been prevented to a large extent*,* but they still exist. When there is overlap*,* some costs are the responsibility of one organization and some of another organization. Maybe nothing special will happen*,* but well*,* at some point*,* the insured will abuse*,* and the percentage of insured and the insurance payer will be different*,* especially when we have free insurance” (P:45).*

***Injustice caused by the variation in service packages of different insurance organizations***

*“The service package provided in the village is different from the city level in terms of its extent*,* availability*,* and cost*,* and it is not provided anywhere except the village” (P: 6).*

**Table 3:** Challenges and strategies in the financing function of Iran’s insurance ecosystem

Main theme	Subtheme	Challenges	Strategies
**Collecting financial resources**	Insurance premium	• Imbalance between cost and income of insurance organizations • Income arrears of insurance organizations regarding insurance premiums • Unstable financial resources • Challenges of collecting insurance premiums in a centralized and decentralized manner	• Creating a break-even point and balance between costs and expenses • Creating stable financial resources for the country’s insurance system • Realization of insurance premiums received
	Direct Payment	•Reduction of financial protection following the increase of direct payments	• Severing the financial relationship between the patient and the provider by using the function of supplementary insurances
	Subsidy and tax	• Government debts to insurance organizations	• Tax on harmful goods as part of the health sector’s financial resources
**Accumulation**	Accumulation of financial resources	•The low share of accumulation by insurances in the health financing system	• Creating more accumulation and creating cross-subsidy by consolidating funds
**Purchase of service and allocation of financial resources**	Service tariff and pricing	• Multi-tariff services • Unreality of service prices and tariffs in the insurance system	• Unification and actualization of the tariff and price of services through the modification of the tariff process
	Strategic purchase	•Defects in the correct execution of the strategic purchasing process	• Strengthening the role of insurance as a broker in the strategic purchase of services • Separation of the service buyer from the provider
	payment system	• Late payment of insurances to medical institutions	• Reforming the payment system
	Debts of the insurance organization	• Indebtedness of insurance organizations to contracting party institutions • Being the same buyer and service provider	• Management of costs to prevent waste of resources by the government • Development and implementation of clinical guidelines and treatment protocols
	Package of services covered by insurance	• Injustice caused by the variation in the service packages of different insurance organizations	• Review and improve the basic service package in the same way for all insurance organizations
	Stratification, referral system and family physician	• Failure to comply with the referral system by the insured	• Reforming the system of providing and purchasing services with the system of referral, stratification and family physician
	Population covered by insurance	•Overlap of the coverage population due to the lack of a comprehensive and integrated information bank of the insured	•Creating an integrated database system of the population covered by all insurance funds
	Insurance contracts	• Insurance deductions based on contracts	• Paying attention to and respecting the rights and interests of the parties in insurance contracts

#### Strategies

***Creating sustainable financial resources for the insurance system***

*“If we want to create a wonderful future for health insurance*,* it should be planned for the financial resources of health insurance. It means that a financial source should be considered for insurance through the parliament or the government*,* which does not change with the change of the government or the parliament. Every year*,* paying attention to the increase in prices and the budget of this organization should be increased so that the insurance managers can plan for the coming years by relying on the approved budget logically” (P: 23).*

***Establishing a break-even point and balance between costs and expenses***

*“Resources in the health insurance system are determined based on the conditions of the country and people*,* and the expenses are determined upon the needs of the insured and the taste of doctors and diagnostic and medical service providers. For this reason*,* we never see a balance between these two cases. From these conditions*,* we should define the insurance premium under the service package and tariffs or the service package and tariffs under the insurance premium to control this situation” (P: 19).*

***Unification and actualization of service tariffs and prices***

*“We must use the legal potential to make real medical tariffs. Suppose something wants to be added to the organization’s obligations. In that case*,* the tariff expert groups will calculate the effect on the per capita*,* tariff*,* and financial burden. A positive step can be taken by making the tariff prices realistic” (P: 14).*

***Payment system reform***

*“The reform of the payment system*,* from the efficiency to the capita*,* will organize the receipts of the doctors in the outpatient department. I think that the basis for the reform of the payment system in the inpatient department has been provided*,* and we should find a shift from the efficient payment system to the DRG” (P: 2).*

***Development and implementation of clinical guidelines***

*“Guidelines have positive points. On the one hand*,* their positive aspect is in treating patients and providing services*,* which can cause a relative reduction in treatment and costs that go back to insurance. First*,* health should be with us as a policymaker in health and treatment to give the appropriate treatment protocol*,* timing of receiving services*,* treatment methods*,* and medical drugs. The development of protocols should be national so the whole country knows its duty and everyone has the same benefit” (P: 45).*

***Review and improve the basic service package***

*“One of the most important tools is the review of the basic service package of the organization. I quickly reviewed the basic service package of the organization by going through the relevant steps” (P: 15).*

***Reforming the system of providing services with the system of referral and family physician***

*“One of these ways is the family physician and the referral system*,* and when the referral system is launched*,* insurance services will certainly be provided efficiently. Discussing the family physician and the referral system is a positive necessity that all experts agree*,* depending on the condition that it is done completely and comprehensively” (P: 39).*

## Discussion

Previous studies on the challenges of health insurance were limited in scope, focusing on specific issues or individual insurance organizations. As a result, the broader challenges of the health insurance system were not thoroughly examined from a macro and general perspective. This study aims to address this gap by taking an ecosystem approach to analyze the challenges of Iran’s health insurance system and propose strategies to overcome them. The study identified the most significant challenge in Iran’s health insurance ecosystem is the presence of conflicting interests among stakeholders, leading to fragmentation and the establishment of multiple structures that operate independently in terms of supervision and financing. To address this challenge, it is recommended to consolidate the insurance system to a unified entity, prioritize health- oriented services over treatment-oriented ones, and establish a more cohesive and integrated approach to healthcare. In addition, in order to strengthen the governance of the country’s health insurance ecosystem, the number of actors with multiple roles should be reduced and the roles of the actors should be clarified and separated in order to prevent conflicts of interest and structural corruption in this ecosystem.

In the following, the results will be discussed based on the two functions of Stewardship and financing:

### Stewardship Function

Conflict of interest represents a severe challenge to healthcare systems, and the results of the present study and other studies confirm this matter [28, 29]. In the Iranian health insurance ecosystem, multiple insurance funds and organizations with different structures and rules are one factor that leads to conflicts of interest [29]. In addition, actors with various roles, such as the Social Security Organization (SSO), which acts as both a buyer (insurer) and a provider, can cause conflicts of interest and prevent a strategic purchase [30]. Physicians taking on the care provider and policy maker role can also lead to conflicts of interest [16, 29]. The financial resources pooling system of Iran’s health system is inconsistent [31], and the public sector alone has three central insurance funds, including social security, health insurance, and AFMSIO. Meanwhile, banks, oil companies, and other organizations also have individual funds. The World Health Organization (WHO) considers creating multiple insurance funds as one indicator of a breakdown in the organization [27]. The multiplicity of funds reduces the ability of insurance companies to use leverage in strategic purchases. Many buyers who compete with each other reduce the motivation of providers to improve quality and performance [32]. WHO has always emphasized the pooling of insurance funds in its recommendation packages for universal health coverage in countries. Consolidating insurance funds, besides transparency and specifying the people covered by insurance, can also be the basis for improving justice and equality in using insurance payments when receiving health services. In addition, the consolidation of funds helps the health insurance ecosystem reduce disparate funds’ administrative and management costs. However, there is better risk accumulation, and as a result, fund members are more financially protected against financial risks and, at the same time, benefit from the same service coverage [33, 34].

The activities of different insurance funds should be coordinated based on a suitable economic model to correct the processes in the health insurance ecosystem and move towards strategic purchasing. In this model, the input and output of services in the insurance package should be prioritized to preventive services and clinical guidelines. According to the upstream documents, policy and structural integration should be established between different insurance funds to strengthen the integrity and coherence of the health insurance ecosystem. Therefore, there should be political determination and synergy between different actors in the ecosystem to develop quantitative and qualitative coverage of health services and establish strategic purchasing components suitable for the needs of different groups with high risks and costs among the members of society. This important thing requires focusing on short-, medium-, and long-term changes and moving from integrating policy and creating a unified procedure in cost coverage mechanisms and covered services to facilitating the conditions for structural integration [19].

Following the rapid growth of information and communication technologies, the policy-making process has undergone many changes by the governing actors of the ecosystem and has moved from individual wisdom to collective wisdom. Evidence-based policy-making is among the basic approaches to moving from the purely intuitive and unconscious spectrum to the spectrum of scientific logic and considering all the economic, social, political, and cultural aspects of society and organizational capacities [35]. Despite the importance of decision-making based on evidence and collective wisdom among policymakers, this issue has been reported by Salarianzadeh et al. (2019) as one weakness of the governance structure of the MOHME and one of the essential actors in the health insurance ecosystem [36].

In the health insurance ecosystem, accessible integrated human resources, information, and high-quality care are necessary to achieve universal health coverage [37]. Human resources do not have the required managerial and scientific capacity and capabilities in the health insurance ecosystem, and insurance calculation analysis skills, health information management, planning and coordination, budgeting, financial resource management, and decision-based evidence should be strengthened [19]. A transparent, integrated, and robust information system improves the performance of other elements, such as the health insurance ecosystem’s payment and strategic purchasing system.

### Financing Function

Providing financial resources to the health system reduces patients’ financial risk. As a result, most developed countries provide the resources of their health system through taxes or compulsory insurance premiums, so the percentage of payments people make directly is minimal [38]. According to some experts, a situation arises in the absence of transparent and integrated information systems in which people can pay a lower insurance premium by falsely reporting their income in systems where the insurance premium is determined based on the amount of income and causes the balance between the incomes and costs of the insurance organizations in the disturbed ecosystem [39].

In many countries national health insurance ecosystems, the government also pays a percentage of insurance premiums as a subsidy besides paying taxes on the salaries and wages of employees and employers [39]. Government aid and subsidies are sometimes derived from oil revenues and can fluctuate with global oil prices, making them unstable long-term financing sources, as in Iran. Therefore, sustainable financial resources such as taxes (income tax, value-added tax) should form a more significant share of the financial resources of the health insurance ecosystem [40].

One complaint usually made against insurance organizations is the insufficient coverage of the diagnostic and medical costs of the insured and the lack of coverage of health costs and preventive measures. Low insurance premiums are the primary cause of the inability to cover the insured’s health costs; medical expenses of the insured have grown rapidly and disproportionately, there is no optimal allocation and prioritization system, and there are no strategic purchases or services consumed unnecessarily by the insured. The government can achieve universal coverage by allocating sustainable financial resources from the public budget or subsidies to insurance funds and organizations, in addition to rising insurance premiums and other corrective measures to rationalize the consumption pattern and control costs [41].

Insurance premiums are considered one of the primary sources of health financing in most countries [17]. One of the ways that governments can move towards sustainable financing is by collecting income through advance payment and insurance premiums, which is the path most countries close to universal insurance coverage and financing sustainability have chosen [42]. Based on this study’s results, the importance of this issue has been emphasized in Iran’s health insurance laws.

Tariff setting is considered one of the essential policy tools of any country’s health and insurance system, adequate in justice, efficiency, quality, and accountability in the provision of health services. Tariffs in The Iranian health insurance ecosystem have always faced challenges, including unrealistic and out-of-date tariffs and multi-tariff services in the public and private sectors [43]. When the service tariff is not accurate, and there are several cracks in the insurance system, it will not be possible to make a strategic purchase in a real way in the health insurance ecosystem [44]. Despite the power of actors such as doctors, who review the tariffs annually, to determine medical tariffs, there is no transparent process for the pricing of medical services that can effectively manage the conflict of interests of different actors in this field [45]. Tariffs are an essential component of the resource allocation and purchasing process in the health insurance ecosystem and can be used to regulate the relationships between providers, recipients, purchasers of services, and payers and determine the content of the package of services covered by insurance as a guide in the decision to purchase services [43].

One criticism of the tariff system in Iran is the lack of coordination between the decision-makers and the different actors of the health insurance ecosystem. Tariffs in Iran are applied with other logic and forms, leading to a multi-tariff system for services. The Supreme Council of Health Insurance transferred tariff settings to the health system in 2014. There were many conflicts between the medical system and the insurance organizations during the implementation of this law, increasing private sector service tariffs and out-of-pocket expenses [45]. The pricing of diagnostic and therapeutic services in the private sector was again referred to SCHI and the Cabinet in 2016.

Creating more capacity to use the strategic purchase of health services is necessary to promote and improve the financing function of the country’s health insurance ecosystem. Strategic purchases lead to a significant increase in the efficiency of the country’s health system. Increasing concerns about the sustainability of the financing system have been raised in recent years as the service package and insurance coverage have reached an almost universal level. Strategic purchasing will play a key role in improving the health insurance ecosystem.

Iran’s payment system is complex, and the contracts signed by insurers with providers do not consider the price, quantity, or quality of services provided. The payment methods currently used as a payment system for providers have led to adverse incentives for providers, including over-providing services, increasing costs, and enhancing the deficit of insurance funds. Therefore, the payment system should be revised to balance the financial risk between providers and buyers and use payment mechanisms that have better incentives to increase efficiency, justice in access, consumer satisfaction, and service quality [44, 46]. This issue was also emphasized by the interviewees.

Health insurance ecosystem goals can only be reached through prevention and health service coverage in the framework of referral systems and family doctor programs in some countries [47]. Implementing the family doctor program in the health insurance ecosystem involves two actors, MOHME and IHIO, and managers at different levels of these two organizations must cooperate and coordinate for the program to move correctly and organize [48]. Currently, only IHIO and SSO insureds have entered the urban family doctor program, and other insureds (such as AFMSIO and banks.) have not entered this plan for various reasons; this defect causes the referral system to be bypassed. As a result, the country’s health insurance ecosystem should be modified to ensure a single insurance policy and consolidation of insurance [49]. While many laws, especially those concerning development programs, have been approved, how health services are purchased does not follow the strategic purchasing method in the country. Through the contract with the service providers, insurance organizations pay the costs after delivering the service to the insured and after a long delay to the provider. No control and supervision are applied to the purchase process in such a mechanism [50]. A clinical guideline can be used as a governance tool to control costs, clarify the strategic procurement process, manage service providers’ work, and evaluate their quality of service in insurance contracts [51]. Updating the service package and prioritizing packages covered by insurance should be considered. For example, in the coverage of services, the insurance company should specify the priority of services. Currently, insurances only cover a fixed percentage of expenses and do not distinguish much between high-cost and low-cost services [32].

This study was conducted during COVID-19, when it was impossible to conduct face-to-face interviews with several participants because of restrictions on intercity travel. For this reason, interviews with these people were conducted virtually.

## Conclusion

The essential challenge of supervision in Iran’s health insurance ecosystem is the conflict of interests among ecosystem actors, which has led to the fragmentation of multiple structures in Iran’s health insurance system and the creation of a gap between supervision, financing, and insular functioning of the ecosystem. A lack of consensus exists among actors regarding the role of insurance regarding the method of collecting insurance premiums, the amount of the deductible, and the package of covered services. This issue has caused the balance between insurance obligations and insurance premiums to be disturbed. Using multiple information systems is a solution to create transparency and uniformity in implementing insurance laws and guidelines. Establishing communication between multiple systems to implement the electronic prescribing system and the electronic health record has caused challenges in the health insurance ecosystem. Due to the expansion of the use of new insurance technologies in the health insurance ecosystem, moving towards the electronicization of insurance processes is on the agenda of insurance organizations. Also, by creating an integrated monitoring system of fund databases of insurance organizations and creating transparency of integrated information and virtual aggregation of multiple insurance funds, an effective step can be taken to solve the challenges of the health insurance ecosystem. Among other effective reforms that lead to the creation of an integrated and evidence-based decision-making system in the health insurance ecosystem, there will be the development of clinical guidelines, the modification of the payment system and the updating of the service tariff system, and the revision of the basic service package of insurance organizations.

In the end, according to the results of the present study, policy recommendations for policymakers of the health system and the health insurance system are presented in order to deal with the major challenges mentioned in this study:

1. Creating a single stewardship system in the insurance ecosystem: It is suggested to create a coherent insurance system through a single stewardship system of health insurance, and by providing and paying more attention to health-oriented services, instead of being treatment-oriented, the health insurance ecosystem has become a health-oriented system. In such a situation, in addition to cost management, the amount of direct payments can also be reduced.
2. Reducing the multiplicity of roles of actors in the insurance ecosystem: It is suggested that in order to strengthen the governance of the country’s health insurance ecosystem, the number of actors who have multiple roles should be reduced and the roles of the actors should be clear and separated in order to prevent conflicts of interest and structural corruption.
3. Strengthening and increasing the capacity to use new roles in the health insurance ecosystem: The entry and emergence of actors with the role of knowledge and technology mediators and brokers in the health insurance ecosystem can be an effective factor in strengthening governance and filling the structural gaps between the actors of the health insurance ecosystem, and therefore, it is suggested to create the necessary capacity to enter and use the capabilities of such actors.

## Electronic supplementary material

Below is the link to the electronic supplementary material.

Supplementary Material 1

## Data Availability

The data analyzed is available from the corresponding author on reasonable request.

## Abbreviations

UHI: Universal Health Insurance
MoHME: Ministry of Health and Medical Education
MSIO: Medical Services Insurance Organization
SSO: Social Security Organization
IHIO: Iran Health Insurance Organization
AFMSIO: Armed Forces Medical Services Insurance Organization
WHO: World Health Organization
