# Supplementary material for: Iran’s health insurance ecosystem: challenges and strategies
Source: BMC Public Health. 2024 Sep 11;24:2470. doi: 10.1186/s12889-024-19998-2 (PMC11389450; doi:10.1186/s12889-024-19998-2)
Supplement: Supplementary file 1 — Supplementary Material 1 [file 12889_2024_19998_MOESM1_ESM.docx]

***Interview framework***

**Interviewer information:**

First and last name:

Gender:

Organizational position:

Geographical scale:

**In your opinion:**

1. What are the challenges of Iran's health insurance ecosystem?
2. What are the strategies to deal with these challenges?
